# Successful extracorporeal cardiopulmonary resuscitation with percutaneous coronary intervention after nearly three hours of refractory out-of-hospital cardiac arrest: a case report

**DOI:** 10.1016/j.resplu.2026.101357

**Published:** 2026-05-06

**Authors:** Rongheng Lu, Aijun Shan, Shaoxi Chen

**Affiliations:** Accident and Emergency Department, The University of Hong Kong-Shenzhen Hospital, Shenzhen 518053, China

**Keywords:** Extracorporeal cardiopulmonary resuscitation, Prolonged cardiac arrest, Extracorporeal membrane oxygenation, Percutaneous coronary intervention, Out-of-hospital cardiac arrest, Case report

## Abstract

**Background:**

Extracorporeal cardiopulmonary resuscitation (ECPR) has emerged as a rescue strategy for refractory cardiac arrest. However, evidence supporting ECPR after extremely prolonged cardiopulmonary resuscitation (CPR) exceeding 150 min in non-hypothermic cardiac arrest remains exceedingly rare, and the upper time limit for initiating ECPR has not been definitively established.

**Case summary:**

A 52-year-old previously healthy male experienced out-of-hospital cardiac arrest (OHCA) with an initial rhythm of asystole that subsequently converted to a shockable rhythm. Following approximately 174 min of resuscitation with intermittent unstable return of spontaneous circulation (ROSC), venoarterial extracorporeal membrane oxygenation (VA-ECMO) was emergently initiated. The initial arterial pH was 7.083 with a lactate of 17.54 mmol/L. Coronary angiography revealed 95% stenosis of the proximal left anterior descending artery (LAD) with visible thrombus, and percutaneous coronary intervention (PCI) with drug-eluting stent implantation was performed under ECMO support, achieving TIMI grade 3 flow. An intra-aortic balloon pump (IABP) was placed for additional hemodynamic support. The patient developed acute kidney injury requiring continuous renal replacement therapy (CRRT). He was successfully weaned from ECMO on day 3, regained consciousness on day 4, was extubated on day 5, and was discharged on day 31 with a Cerebral Performance Category (CPC) score of 1 and a left ventricular ejection fraction (LVEF) of 49%. At 12-month follow-up, the patient remains in excellent health.

**Discussion:**

To our knowledge, this is one of the longest reported durations from cardiac arrest to ECMO initiation (∼174 min) in a non-hypothermic OHCA patient who achieved complete neurological recovery (CPC 1). This case challenges the prevailing paradigm that resuscitation beyond 60 min is futile and underscores the potential of ECPR combined with timely revascularization when favorable prognostic factors coexist.

## Introduction

Cardiac arrest remains a leading cause of mortality worldwide, with approximately 350,000 out-of-hospital cardiac arrests (OHCA) occurring annually in the United States alone.^1^ Survival rates after OHCA remain dismally low, typically under 10%, with substantial regional variation.^2^ The likelihood of neurologically favorable survival declines progressively with increasing duration of cardiopulmonary resuscitation (CPR), falling below 5% if return of spontaneous circulation (ROSC) is not achieved within 30–45 min of standard advanced cardiac life support (ACLS).^3^, ^4^

Extracorporeal cardiopulmonary resuscitation (ECPR)—the emergent initiation of venoarterial extracorporeal membrane oxygenation (VA-ECMO) during refractory cardiac arrest—has emerged as a promising rescue intervention that restores organ perfusion and provides a critical time window for the treatment of reversible underlying etiologies.^5^, ^6^ The Extracorporeal Life Support Organization (ELSO) consensus statement recommends considering ECPR after 10–20 min of failed conventional resuscitation, particularly when a reversible cause is suspected.^7^ Three landmark randomized controlled trials—the ARREST trial,^8^ the Prague OHCA trial,^9^ and the INCEPTION trial^10^—have provided pivotal, albeit nuanced, evidence regarding the efficacy of ECPR in refractory OHCA, with pooled analyses demonstrating improved neurologically favorable survival in patients with shockable rhythms treated at experienced, high-volume centers.^11^

However, the upper time limit for initiating ECPR remains undefined. The ELSO consensus acknowledges neurologically intact survival after over 3 h of CPR before ECPR in hypothermic arrests, while noting that in non-hypothermic settings, the majority of survivors were established on ECMO within 60 min of arrest onset.^7^ Among the longest reported non-hypothermic cases, Wang et al. described a patient who underwent 152 min of CPR and achieved full neurological recovery following ECPR combined with continuous renal replacement therapy (CRRT).^12^ The University of Minnesota ECPR cohort demonstrated that ECPR-facilitated survival remained possible after 98 min of CPR, though neurologically favorable survival declined by approximately 25% with every 10 min of delay beyond 29 min.^3^

Herein, we present the case of a 52-year-old male with OHCA who underwent approximately 174 min of resuscitation before successful ECPR initiation, followed by emergent percutaneous coronary intervention (PCI) with stent implantation under ECMO support, ultimately achieving survival with complete neurological recovery (CPC 1) and excellent 12-month follow-up. To our knowledge, this represents one of the longest reported durations from non-hypothermic cardiac arrest to ECMO initiation with full neurological recovery, providing compelling evidence that CPR duration alone should not serve as an absolute contraindication to ECPR when favorable prognostic factors are present.

## Case presentation

This case report was prepared in accordance with the CARE (CAse REport) guidelines.^13^ Written informed consent was obtained from the patient for publication of this case report and any accompanying images.

### Patient information and presenting concerns

A 52-year-old male with no significant past medical history experienced sudden collapse on January 20, 2025. At approximately 22:17, while at work, the patient complained of abdominal discomfort lasting approximately 30 min. He collapsed and lost consciousness after returning from the restroom. A colleague witnessed the event and immediately initiated manual chest compressions while calling for emergency medical services (EMS) at 22:17.

### Pre-hospital management

In the Shenzhen EMS system, ambulance crews are staffed with a physician (typically emergency medicine-trained), a registered nurse, and a driver—a model that differs from many Western EMS systems where paramedics deliver prehospital care. Upon arrival, the EMS physician immediately assumed resuscitation leadership, taking over manual chest compressions from the bystander, establishing intravenous access, administering intravenous epinephrine, and providing bag-valve-mask (BVM) ventilation. Manual chest compressions were maintained throughout ambulance transport. The patient arrived at the Emergency Department (ED) of The University of Hong Kong-Shenzhen Hospital at 22:34, approximately 17 min after the witnessed collapse. Bystander CPR was initiated immediately after collapse, minimizing the no-flow interval.

### Emergency department resuscitation

Upon ED arrival, the patient had no palpable pulse, no measurable blood pressure, and no spontaneous respirations. Body temperature was 34.9°C (spontaneous hypothermia). Bilateral pupils were dilated to 6 mm and non-reactive to light. Manual chest compressions were continued and the patient was endotracheally intubated to secure the airway. A mechanical chest compression device was subsequently applied to ensure consistent CPR quality during the prolonged resuscitation. When the mechanical device was not in use, team-based manual compressor rotation at 2-min intervals was employed. Intravenous access was established, and the cardiology and coronary care unit (CCU) teams were emergently consulted.

The initial monitored rhythm was asystole. Advanced cardiac life support (ACLS) was performed per protocol, including intravenous epinephrine (1 mg per dose, administered at regular intervals, ultimately totaling 30 mg over the resuscitation course) and sodium bicarbonate for severe metabolic acidosis. Over the course of resuscitation, the rhythm evolved from asystole to a shockable rhythm, and four direct-current defibrillation attempts (200 J biphasic each) were delivered at 22:44, 22:52, 23:06, and 23:12. Intravenous amiodarone 300 mg was administered at 23:06 for refractory ventricular fibrillation. Vasopressor support with dopamine was also initiated.

Serial bedside transthoracic echocardiography (point-of-care ultrasound, POCUS) was used throughout the resuscitation to monitor cardiac contractility and the presence or absence of organized cardiac activity, as invasive arterial blood pressure monitoring was not established until ECMO cannulation at 01:11. Prior to arterial line placement, pulse assessment relied on manual palpation supplemented by echocardiographic visualization.

The following is a detailed chronological account of the circulatory status during resuscitation (Table 1): From 22:34 to 23:29 (55 min), continuous CPR was performed without detectable ROSC; the rhythm evolved from asystole to ventricular fibrillation, and four defibrillation attempts were delivered. Between 23:29 and 23:36 (7 min), bedside echocardiography revealed cardiac contractions, but no pulse was palpable; CPR was continued. From 23:36 to 00:02 (26 min), CPR continued without palpable pulse. Between 00:02 and 00:11 (9 min), palpable pulse was present; bedside echocardiography at 00:02 demonstrated an LVEF of 47%, and a 12-lead electrocardiogram (ECG) showed ST-segment elevation consistent with acute anterior STEMI. Between 00:11 and 00:21 (10 min), the pulse was lost and CPR was resumed. From 00:21 to 00:26 (5 min), palpable pulse was again present. At 00:26, the ECPR team was activated. Between 00:34 and 00:58 (24 min), the pulse was lost again and continuous CPR was performed. From 00:58 to 01:11 (13 min), dopamine infusion achieved a weak blood pressure of 88/60 mmHg. At 01:11, VA-ECMO was initiated.

**Table 1:** Timeline of key events.

**Date/time**	**Min from arrest**	**Event**	**Circulation status/key parameters**
Jan 20, 22:17	0	Witnessed cardiac arrest	Collapse; bystander CPR initiated immediately
22:17–22:34	0–17	Bystander → EMS CPR	EMS: physician-led; BVM ventilation; IV epi; manual CPR during transport
22:34	∼17	ED arrival	Asystole; T 34.9°C; pupils 6 mm; intubation; mechanical + manual CPR
22:44	∼27	1st defibrillation (200 J)	Rhythm evolved: asystole → VF
22:52	∼35	2nd defibrillation (200 J)	Continuous CPR; epi 1 mg IV at regular intervals
23:06	∼49	3rd defib; amiodarone 300 mg	Refractory VF
23:12	∼55	4th defibrillation (200 J)	Ongoing hemodynamic instability
23:29–23:36	∼72–79	Bedside echo: cardiac contractions	No palpable pulse; CPR continued (pseudo-PEA?)
23:36–00:02	∼79–105	Continuous CPR	No palpable pulse
00:02–00:11	∼105–114	ROSC: palpable pulse	Echo LVEF 47%; ECG: anterior STEMI identified
00:11–00:21	∼114–124	Pulse lost; CPR resumed	No palpable pulse
00:21–00:26	∼124–129	ROSC: palpable pulse	Hemodynamically unstable
00:26	∼129	ECPR team activated (2nd consult)	pH 7.083, lactate 17.54 mmol/L; total epi 30 mg
00:34–00:58	∼137–161	Continuous CPR	No palpable pulse; BP unrecordable
00:58–01:11	∼161–174	Dopamine infusion	Weak BP 88/60 mmHg (vasopressor-dependent)
01:11	∼174	VA-ECMO initiated	R fem vein 23 Fr, L fem artery 17 Fr; arterial line placed
01:35	∼198	Coronary angiography	LM patent; LAD prox 95% stenosis + thrombus
02:30–02:50	∼253–273	PCI: 1 DES to LAD	TIMI-3 flow; TnT peak 63.19 ng/mL
10:05	∼708	IABP placed	Persistent circulatory instability
19:00	Day 1	CRRT initiated	AKI with anuria
Jan 23, 18:30	Day 3	ECMO decannulated	BP 106/48; sinus rhythm, HR 100; LVEF 45%
Jan 24	Day 4	Neurological improvement	GCS E3VTM6; follows commands
Jan 25	Day 5	Extubated	Spontaneous breathing adequate
Jan 26	Day 6	Fully conscious	GCS 15 (E4V5M6); conversant, oriented
Jan 28	Day 8	IABP removed	Hemodynamically stable without support
Feb 6	Day 17	Echocardiography	LVEF improved to 49%
Feb 20	Day 31	Discharged	CPC 1; no neurological deficits
Feb 11, 2026	12 mo	Follow-up	Excellent health; full functional recovery

AKI, acute kidney injury; BP, blood pressure; BVM, bag-valve-mask; CPR, cardiopulmonary resuscitation; CPC, Cerebral Performance Category; CRRT, continuous renal replacement therapy; DES, drug-eluting stent; ECG, electrocardiogram; ECMO, extracorporeal membrane oxygenation; ECPR, extracorporeal cardiopulmonary resuscitation; ED, emergency department; EMS, emergency medical services; epi, epinephrine; GCS, Glasgow Coma Scale; HR, heart rate; IABP, intra-aortic balloon pump; IV, intravenous; LAD, left anterior descending artery; LM, left main; LVEF, left ventricular ejection fraction; PCI, percutaneous coronary intervention; PEA, pulseless electrical activity; ROSC, return of spontaneous circulation; STEMI, ST-elevation myocardial infarction; T, temperature; TIMI, Thrombolysis in Myocardial Infarction; TnT, troponin T; VA, venoarterial; VF, ventricular fibrillation.

In total, identifiable intervals of palpable spontaneous pulse comprised approximately 14 min (00:02–00:11 and 00:21–00:26), with an additional 13 min of weak perfusion supported by vasopressors (00:58–01:11) and 7 min of echocardiographic cardiac activity without palpable pulse (23:29–23:36). Initial arterial blood gas analysis revealed severe metabolic acidosis with a pH of 7.083 and a markedly elevated lactate level of 17.54 mmol/L, reflecting severe and prolonged tissue hypoperfusion.

### Decision to initiate ECPR

The decision to initiate ECPR evolved over the course of the resuscitation and involved two separate consultations with the ECMO team. The first consultation was requested at approximately 40 min into the resuscitation. At that time, the patient had bilateral fixed dilated pupils, persistent asystole, and no signs of ROSC. The ECMO team assessed the patient and determined that ECPR was not indicated based on these unfavorable prognostic signs.

However, as resuscitation continued, the clinical picture evolved favorably: the rhythm converted from asystole to a shockable rhythm, intermittent episodes of ROSC were observed, bilateral pupils constricted from 6 mm to 4 mm with restored light reflex, and spontaneous limb movements emerged. Critically, a 12-lead ECG obtained during an episode of ROSC revealed acute anterior STEMI, identifying a clearly reversible etiology amenable to catheter-based intervention. These developments prompted a second ECMO team consultation at 00:26 on January 21 (approximately 129 min after the initial cardiac arrest). Based on the convergence of favorable prognostic factors—(1) witnessed cardiac arrest with immediate bystander CPR; (2) rhythm evolution from asystole to a shockable rhythm with intermittent unstable ROSC suggesting viable myocardium; (3) pupillary constriction and spontaneous limb movements indicating preserved brainstem function; (4) identification of acute anterior STEMI amenable to PCI; (5) relatively young age (52 years) with no significant comorbidities; and (6) ongoing high-quality CPR (both manual and mechanical)—the decision to proceed with ECPR was made.

Several factors contributed to the prolonged interval from cardiac arrest to ECMO initiation. The arrest occurred at 22:17 (nighttime), and the ECPR team operates on an on-call basis, requiring mobilization from off-site. The initial ECMO consultation at ∼40 min was negative, and the evolving clinical picture only prompted the second consultation at ∼129 min. Additionally, in the Chinese medical system, family informed consent is required before initiating ECPR, which necessitated time for the family to understand the clinical situation, prognosis, and associated risks (Table 2).

**Table 2:** Comparison with published cases of prolonged CPR and ECPR.

**Parameter**	**Present case**	**Wang et al. (2025)**^12^	**Chen et al. (2025)**^14^	**UMN cohort**^3^
Age/sex	52/Male	53/Male	Middle-aged/Male	Cohort (*n* = 160)
Setting	OHCA	OHCA	OHCA	OHCA (VF/VT)
Initial rhythm	Asystole → VF	VF	Not specified	VF/VT
Bystander CPR	Yes	Yes (exercise)	Not specified	Variable
CPR to ECMO	∼174 min	∼152 min	>150 min	Max ∼98 min
Intermittent ROSC	Yes (multiple)	Not reported	Not reported	Variable
pH (initial)	7.083	Not reported	Not reported	Progressive ↓
Lactate (mmol/L)	17.54	Not reported	Not reported	Progressive ↑
Epinephrine total	30 mg	Not reported	Not reported	Variable
Temperature at ED	34.9°C	Not reported	Not reported	Variable
Reversible cause	LAD STEMI (95%)	Not specified	LAD stenosis	Cardiac
PCI	Yes (1 DES)	No	Yes	Most patients
IABP	Yes	No	Not reported	Variable
ECMO duration	∼65 h	Not reported	Not reported	Variable
CRRT	Yes (AKI)	Yes	Not reported	Not reported
TTM	No (T 34.9°C at arrival)	Yes	Not specified	Variable
Consciousness	Day 4	Day 7	Not specified	Variable
CPC at discharge	1	1	Alive	Variable
Follow-up	12 mo, excellent	2 wk, full recovery	Discharged	≬6 mo

AKI, acute kidney injury; CPC, Cerebral Performance Category; CPR, cardiopulmonary resuscitation; CRRT, continuous renal replacement therapy; DES, drug-eluting stent; ECMO, extracorporeal membrane oxygenation; ED, emergency department; IABP, intra-aortic balloon pump; LAD, left anterior descending artery; OHCA, out-of-hospital cardiac arrest; PCI, percutaneous coronary intervention; ROSC, return of spontaneous circulation; STEMI, ST-elevation myocardial infarction; T, temperature; TTM, targeted temperature management; UMN, University of Minnesota; VF, ventricular fibrillation; VT, ventricular tachycardia.

### ECPR initiation and ECMO management

VA-ECMO was successfully initiated at 01:11 on January 21, approximately 174 min after the initial cardiac arrest. Peripheral cannulation was performed percutaneously using the Seldinger technique via the right femoral vein (23 Fr drainage cannula) and left femoral artery (17 Fr return cannula). Invasive arterial blood pressure monitoring was established simultaneously. Chest compressions were discontinued immediately upon confirmation of adequate ECMO flow and hemodynamic stabilization.

A notable echocardiographic finding was recorded during the resuscitation: bedside transthoracic echocardiography at 00:02 on January 21 (during an episode of ROSC) demonstrated an LVEF of 47%, indicating that despite the prolonged arrest, significant myocardial contractile function was preserved. Serial echocardiographic assessments under ECMO support subsequently showed an initial decline in LVEF (no detectable wall motion at 12:06 on January 21, recovering to 45% by January 22), followed by gradual improvement to 49% by February 6 (Table 3).

**Table 3:** Serial Echocardiographic Assessments.

**Date/time**	**Hospital day**	**Condition**	**LVEF**	**Comments**
Jan 21, 00:02	Day 1	Intermittent ROSC, pre-ECMO	47%	Preserved contractility despite prolonged arrest
Jan 21, 12:06	Day 1	On ECMO, post-PCI	No detectable wall motion	Likely myocardial stunning
Jan 22, 15:58	Day 2	On ECMO	45%	Early recovery of contractility
Jan 23	Day 3	On ECMO, pre-decannulation	45%	Stable; supported ECMO weaning decision
Jan 27	Day 7	Post-ECMO, post-IABP removal	40–45%	Mild reduction; recovering
Feb 6	Day 17	Ward	49%	Continued improvement; favorable remodeling

ECMO, extracorporeal membrane oxygenation; IABP, intra-aortic balloon pump; LVEF, left ventricular ejection fraction; PCI, percutaneous coronary intervention; ROSC, return of spontaneous circulation.

### Coronary angiography and PCI

The patient was transferred to the cardiac catheterization laboratory at 01:35 under ongoing VA-ECMO support. Emergent coronary angiography revealed a patent left main coronary artery, critical 95% stenosis of the proximal left anterior descending artery (LAD) with visible thrombus, and no hemodynamically significant lesions in the left circumflex artery (LCx) or right coronary artery (RCA). The proximal LAD lesion was identified as the culprit responsible for the acute anterior STEMI.

PCI was performed on the LAD lesion between approximately 02:30 and 02:50. Balloon pre-dilation was performed, followed by successful deployment of one drug-eluting stent, achieving TIMI grade 3 flow (complete antegrade reperfusion) with no residual stenosis. Peak cardiac troponin T (TnT) reached 63.19 ng/mL, reflecting the extensive myocardial injury sustained during the prolonged arrest.

An intra-aortic balloon pump (IABP) was subsequently inserted at 10:05 on January 21 to provide additional hemodynamic support for persistent circulatory instability despite ECMO. The IABP augmented diastolic coronary perfusion pressure and reduced left ventricular afterload, complementing the VA-ECMO circuit in supporting myocardial recovery.

### Post-resuscitation intensive care

**Hemodynamic management:** Following PCI, the patient’s circulation remained unstable initially but gradually improved. By January 23, blood pressure had stabilized at 106/48 mmHg with sinus rhythm at 100 beats per minute. VA-ECMO was successfully weaned and decannulated at 18:30 on January 23 (total ECMO support duration: approximately 65 h). The IABP was removed on January 28 (hospital day 8) after confirmation of hemodynamic stability without mechanical circulatory support.

**Renal support:** The patient developed acute kidney injury (AKI) with anuria, likely secondary to prolonged hypoperfusion during the cardiac arrest and subsequent cardiogenic shock. Continuous renal replacement therapy (CRRT) was initiated at 19:00 on January 21 for volume management, metabolic optimization, and solute clearance. Intermittent CRRT sessions were continued through January 24. Renal function subsequently recovered with return of adequate urine output.

**Temperature management and neurological recovery:** Body temperature at ED arrival was 34.9°C (spontaneous hypothermia). Active targeted temperature management (TTM) was not implemented; fever prevention was maintained per standard post-resuscitation care protocols. Neurological recovery was remarkably favorable. On January 24 (day 4), under light sedation and analgesia, the patient was able to open eyes to voice and follow commands (grip hands on request), with a Glasgow Coma Scale (GCS) score of E3VTM6 (9T, intubated). On January 25 (day 5), the endotracheal tube was removed, and the patient was successfully weaned from mechanical ventilation. By January 26 (day 6), the patient was fully conscious and alert, engaging in conversation and answering questions appropriately, with a GCS of E4V5M6 (15/15) and no focal neurological deficits.

**Cardiac function:** Serial echocardiographic assessments demonstrated dynamic changes in left ventricular function (Table 3). The initial LVEF of 47% (during intermittent ROSC prior to ECMO) declined transiently, with no detectable wall motion at 12 h post-arrest under ECMO support, likely reflecting myocardial stunning. LVEF recovered to 45% by January 22 and ultimately improved to 49% by February 6, suggesting favorable myocardial remodeling following successful revascularization.

### Outcome and follow-up

The patient’s clinical course was characterized by steady improvement across all organ systems. He was transferred from the ICU to the general cardiology ward and was discharged on February 20, 2025 (hospital day 31) in good condition. At discharge, his Cerebral Performance Category (CPC) score was 1 (good cerebral performance, normal daily activities), LVEF was 49%, and he had no focal neurological deficits.

At discharge, the patient was prescribed guideline-directed medical therapy including dual antiplatelet therapy, dose-adjusted anticoagulation, high-intensity lipid-lowering therapy, heart rate control agents, and diuretics.

At 12-month follow-up on February 11, 2026, the patient reported excellent health with full return to normal daily activities and no cardiovascular symptoms, readmissions, or neurological complaints.

## Discussion

This case documents the successful resuscitation of a 52-year-old patient with OHCA who underwent approximately 174 min from cardiac arrest to ECMO initiation—including periods of intermittent, profoundly unstable ROSC—followed by emergent PCI and IABP support, ultimately achieving complete neurological recovery (CPC 1) with excellent 12-month follow-up. To our knowledge, this represents one of the longest reported intervals from non-hypothermic cardiac arrest to ECMO initiation with full neurological recovery, exceeding the 152-min case reported by Wang et al.^12^

### Redefining the limits of prolonged resuscitation

The relationship between CPR duration and outcomes is well established. A landmark analysis of nearly 350,000 in-hospital cardiac arrest patients demonstrated that the probability of survival and favorable neurological outcome falls below 1% after 39 and 32 min of CPR, respectively.^15^ In the ECPR context, the University of Minnesota cohort showed a 25% decline in neurologically favorable survival per 10 min of CPR beyond 29 min, with the longest survivor undergoing 98 min of CPR.^3^ Wengenmayer et al. reported 67% survival with 6–20 min of CPR but only 10% with 45–60 min before ECPR.^16^

Our case, along with the 152-min case by Wang et al.^12^ and the 150-min case by Chen et al.,^14^ forms a growing body of evidence suggesting that the salvage window for ECPR may extend well beyond conventionally accepted limits. The ELSO consensus statement explicitly avoids specifying a maximum CPR duration for ECPR candidacy, instead recommending individualized assessment.^7^

### Unique features: from asystole to shockable rhythm with intermittent ROSC

Several distinctive features merit discussion. First, the initial rhythm was asystole, typically associated with substantially worse outcomes.^17^ However, rhythm evolution to VF during resuscitation suggested a dynamic acute coronary syndrome. Second, intermittent unstable ROSC—with multiple episodes of palpable pulse (totaling approximately 14 min) and additional periods of echocardiographic cardiac activity without palpable pulse (pseudo-PEA)—indicated preserved myocardial viability. Debaty et al. demonstrated that signs of life during CPR are significantly associated with favorable neurological outcomes in ECPR patients.^20^

The total epinephrine dose (30 mg) was remarkably high. The favorable outcome despite this suggests that cumulative epinephrine dosing may have different prognostic implications in the ECPR era, where mechanical circulatory support can override the hemodynamic consequences of catecholamine excess.^19^

### ECPR as a bridge to PCI: an integrated approach

The identification of acute anterior STEMI during intermittent ROSC was pivotal. ECPR served as a critical bridge to PCI. The multimodal support strategy (VA-ECMO + PCI + IABP) exemplifies the integrated approach that characterizes successful ECPR programs. A meta-analysis identified PCI as an independent favorable survival predictor in ECPR (OR 1.63).^21^ The sequential de-escalation—ECMO on day 3, IABP on day 8—reflected progressive myocardial recovery.

This case also highlights the importance of establishing 24/7 in-house ECPR capability and early activation protocols. The initial negative ECMO consultation at ∼40 min was clinically appropriate, yet the subsequent favorable evolution underscores the importance of serial reassessment and willingness to reconsider ECPR candidacy.

### Temperature management

Body temperature at ED arrival was 34.9°C (spontaneous hypothermia). Active targeted temperature management (TTM) was not implemented, consistent with current evidence from the TTM2 trial.^22^ The spontaneous hypothermia at presentation may have provided an incidental degree of neuroprotection during the prolonged low-flow state, though no causal inference can be drawn from a single case.

### Acute kidney injury and the role of CRRT

The patient developed AKI with anuria. CRRT was initiated on day 1 for volume management, metabolic optimization, and clearance of inflammatory mediators. Wang et al. highlighted the role of CRRT in their 152-min case.^12^ Renal function recovered during hospitalization.

### Evidence from randomized controlled trials in context

The ARREST trial demonstrated 43% versus 7% survival (*p* = 0.023).^8^ The Prague OHCA trial showed improved long-term survival.^9^, ^18^ The INCEPTION trial found no significant difference (20% vs 16%).^10^ Mean arrest-to-ECMO times across these trials ranged from 59 min to 74 min^23^—substantially shorter than our 174 min.

### Limitations

Several limitations should be acknowledged. First, as a single case report, no causal inference can be drawn, and survivorship bias cannot be excluded. Second, invasive arterial monitoring was not established until ECMO cannulation at 01:11; periods of weak circulation (pseudo-PEA) may have been present but undetected. Third, the ∼174-min interval includes identifiable periods of spontaneous pulse (∼14 min), vasopressor-supported perfusion (∼13 min), and echocardiographic activity without palpable pulse (∼7 min), and should not be interpreted as 174 min of uninterrupted chest compressions. Fourth, body temperature at ECMO initiation was not recorded. Fifth, the resources required are substantial and may not be available in all settings. Sixth, the convergence of multiple favorable factors limits generalizability.

## Conclusion

This case demonstrates that survival with complete neurological recovery (CPC 1) is achievable approximately 174 min after non-hypothermic OHCA, when ECPR is combined with emergent PCI for a reversible cardiac etiology. CPR duration alone should not determine ECPR candidacy; rather, a holistic assessment of favorable prognostic factors—including witnessed arrest with bystander CPR, signs of brainstem viability, rhythm evolution, and suspected reversible etiology—should guide clinical decision-making. Serial reassessment of ECPR candidacy is essential, as the clinical picture may evolve favorably during prolonged resuscitation.

## Learning points/clinical perspectives

1. CPR duration alone should not serve as an absolute contraindication to ECPR; individualized assessment of favorable prognostic factors should guide clinical decision-making, even after resuscitation exceeding 150 min.
2. The evolution of cardiac rhythm from asystole to a shockable rhythm, with intermittent unstable ROSC, may indicate viable myocardium and should prompt serial reassessment of ECPR candidacy rather than termination of resuscitation.
3. An integrated ECPR-to-PCI workflow with multimodal mechanical circulatory support (VA-ECMO + IABP) can achieve complete neurological recovery in acute STEMI-related cardiac arrest, even after extremely prolonged resuscitation.
4. Spontaneous hypothermia at presentation may contribute to neuroprotection during prolonged cardiac arrest. Body temperature should be documented and considered in prognostic assessment.
5. Comprehensive post-resuscitation ICU care, including CRRT for AKI management and serial echocardiographic monitoring to guide mechanical support weaning, is essential for optimizing outcomes.

## Ethics statement

This case report was approved by the Ethics Committee of The University of Hong Kong-Shenzhen Hospital. Written informed consent was obtained from the patient for publication of this case report and any accompanying images.

## Prior presentation

This case has not been previously presented at any conference or meeting.

## Declaration of generative AI in scientific writing

During the preparation of this work, the author(s) used Claude to improve readability and language. After using this tool, the author(s) reviewed and edited the content as needed and take(s) full responsibility for the content of the publication.

## CRediT authorship contribution statement

**Rongheng Lu:** Writing – review & editing, Investigation, Data curation. **Aijun Shan:** Writing – review & editing, Writing – original draft, Visualization, Investigation, Data curation, Conceptualization. **Shaoxi Chen:** Writing – review & editing, Writing – original draft, Visualization, Supervision, Investigation, Data curation, Conceptualization.

## Funding

This research received no external funding.

## Declaration of competing interest

The authors declare that they have no known competing financial interests or personal relationships that could have appeared to influence the work reported in this paper.

## Data Availability

The data supporting the findings of this case report are available from the corresponding author upon reasonable request.
